# Genome-wide identification and characterization of tissue-specific non-coding RNAs in black pepper (*Piper nigrum* L.)

**DOI:** 10.3389/fpls.2023.1079221

**Published:** 2023-03-16

**Authors:** Baibhav Kumar, Bibek Saha, Sarika Jaiswal, U. B. Angadi, Anil Rai, Mir Asif Iquebal

**Affiliations:** Division of Agricultural Bioinformatics, Indian Council of Agricultural Research-Indian Agricultural Statistics Research Institute, New Delhi, India

**Keywords:** circular RNAs (circRNAs), competitive endogenous RNAs (ceRNAs), gene expression regulation, long non-coding RNAs (lncRNAs), spices

## Abstract

Long non-coding RNAs (lncRNAs) and circular RNAs (circRNAs) are the two classes of non-coding RNAs (ncRNAs) present predominantly in plant cells and have various gene regulatory functions at pre- and post-transcriptional levels. Previously deemed as “junk”, these ncRNAs have now been reported to be an important player in gene expression regulation, especially in stress conditions in many plant species. Black pepper, scientifically known as *Piper nigrum* L., despite being one of the most economically important spice crops, lacks studies related to these ncRNAs. From a panel of 53 RNA-Seq datasets of black pepper from six tissues, *namely*, flower, fruit, leaf, panicle, root, and stem of six black pepper cultivars, covering eight BioProjects across four countries, we identified and characterized a total of 6406 lncRNAs. Further downstream analysis inferred that these lncRNAs regulated 781 black pepper genes/gene products *via* miRNA–lncRNA–mRNA network interactions, thus working as competitive endogenous RNAs (ceRNAs). The interactions may be various mechanisms like miRNA-mediated gene silencing or lncRNAs acting as endogenous target mimics (eTMs) of the miRNAs. A total of 35 lncRNAs were also identified to be potential precursors of 94 miRNAs after being acted upon by endonucleases like Drosha and Dicer. Tissue-wise transcriptome analysis revealed 4621 circRNAs. Further, miRNA–circRNA–mRNA network analysis showed 432 circRNAs combining with 619 miRNAs and competing for the binding sites on 744 mRNAs in different black pepper tissues. These findings can help researchers to get a better insight to the yield regulation and responses to stress in black pepper in endeavor of higher production and improved breeding programs in black pepper varieties.

## 1 Introduction

Spices have been an important part of human food and nutrition for thousands of years. Recent studies confirm their diverse significant potentials against various diseases, in addition to their trivial nutritional benefits as functional foods. Black pepper is one of the most important spices containing *piperine* as the active ingredient. Black pepper also contains various volatile oils, oleoresins, and alkaloids, and has a role in suppressing tumor growth and chemoprevention (Butt et al., 2013). Black pepper consumption also improves nutrients absorption and gastrointestinal function. *Piperine*, the main component of black pepper, inhibits the differentiation of fat cells by lowering PPAR (peroxisome proliferator activated receptor) activity and reducing PPAR expression, which is a potential cure for disorders linked to obesity (Park et al., 2012; Negi et al., 2021).

Studies confirming the existence and important role of short (18-23 nucleotides) and long (>200 nucleotides) RNAs over the past two decades have completely changed the view of the cellular mechanism of gene expression. Non-coding regions of the genome (~98% in mammals) are now no longer considered “junk” and are believed to play important regulatory and tissue-specific expression roles (Azlan et al., 2019). LncRNAs are one such important class of ncRNAs with lengths >200 nucleotides and are predominantly found in the cell’s nucleolus, nucleus, or cytoplasm. LncRNAs have little or no coding potential and may contain an ORF only by chance, but are similar to protein-coding mRNAs in aspects like they both are transcribed by RNA polymerase II, spliced and 5′ methyl Guanosine capped and 3′ poly-adenylated. These challenges make the identification and characterization task of lncRNAs a more tedious task (Mattick, 2004; Wang and Chang, 2011).

In a typical mammalian genome, approximately 4%–9% of the genome is transcribed into lncRNAs, which is more than the fraction of the genome that is transcribed into protein-coding mRNAs (~1%) (Amaral et al., 2010). LncRNAs are not actively expressed as in proteins but are involved in a lot of cellular activities. LncRNAs are spread throughout the genome which contains 98%–99% non-coding region and are labeled with respect to the genomic location they are transcribed from, *viz*., those arriving from the intergenic region are called intergenic lncRNAs; intronic lncRNAs are those derived purely from introns, while the exonic lncRNAs are derived from exons of protein-coding genes (Mercer et al., 2009). LncRNAs have been found to be involved in various cellular functions mainly regulating the expression of the genes in *cis*- or *trans*- of their origin. *cis*-Acting lncRNAs may alter the expression of neighboring genes by either blocking the formation of pre-initiation complex (PIC) by attaching to the promoter or interfering the transcription factors or *via* chromatin modifications. The best example of chromatin modification mediated lncRNA function is XIST (X inactive-specific transcript), which is a 19-kb-long human lncRNA which binds to the PRC2 (Polycomb Recessive Complex) to induce H3K27me3 histone modification which leads to transcriptional silencing of genes on the X chromosome. Also, in plants COLDAIR lncRNA (Cold Assisted Intronic Non-coding RNA) works as a necessary repressor of FLC (Flowering Locus C) during vernalization. HOTAIR (Hox Antisense Intergenic RNA) is a trans-acting lncRNA in humans that transports itself to other locations on different chromosomes and regulates the gene expression (Chen and Carmichael, 2010; Song et al., 2016). Apart from the extensive studies done in humans and other mammals, various plant lncRNAs have also been identified including *Arabidopsis thaliana* (Lu et al., 2017), wheat (Lu et al., 2020), rice (Wang et al., 2018; Zhou et al., 2021), maize (Li et al., 2014), tomato (Yang et al., 2019), cucumber (He et al., 2020; Dey et al., 2022), pearl millet (Kumar et al., 2022), and watermelon (Sun et al., 2020). LDMAR (Long Day Specific Male-sterility-associated RNA) which is a 1236 bp long lncRNA can regulate the male sterility sensitive to photoperiod in rice.

Apart from the linear lncRNAs, another important class of non-linear ncRNAs, called circRNAs have emerged, which are formed by the back splicing of 5′ terminus upstream exon of the pre-mRNA with the 3′ downstream exon (Lai et al., 2018). Evidently, circRNAs are more resistant to RNAase degradation due to lack of 5′ cap or 3′ tail-free ends. First reported in 1979 by Hsu, in HeLa cell lines, circRNAs have been studied in many species since then including rice (Lu et al., 2015), wheat (Ren et al., 2018), cucumber (Mu et al., 2016; Zhu et al., 2019), chickpea (Dasmandal et al., 2020) mango (Yang et al., 2022), and watermelon (Sun et al., 2020). Chu et al. (2018) showed that higher expression of the circRNAs has a down regulating effect on its parental genes. Cucumber circRNAs study showed that the circRNAs can also act as miRNA sponges and have a miRNA-circRNA-mRNA network relationship of gene regulation mechanism.

Black pepper (*Piper nigrum* L.) is one of the most widely grown and traded spices in the world and is recognized as the king of spices (Nair et al., 1993). Following the release of the reference genome of black pepper, a lot of studies related to genes of black pepper have been done but no study has been performed till now of the ncRNAs, except for the small miRNAs (Ding et al., 2021), where 128 mature miRNAs and their 1007 target mRNAs were identified. Our work is the first such study to unravel the characteristics and functional roles of larger ncRNAs in black pepper along with circRNAs. For this study, extensive retrieval of black pepper molecular data was made to fetch 53 raw RNA-Seq datasets comprising of >1.2 billion reads covering eight BioProjects and six tissues (flower, fruit, leaf, panicle, root and stem) from 6 black pepper cultivars across four countries, followed by reference transcript assembly for the identification of black pepper lncRNA and circRNAs. This study also aims at having an insight on the tissue-specific nature of lncRNAs and circRNAs and their relationship with miRNAs as competitive endogenous RNAs (ceRNAs), their functional roles in various pathways, development of a freely accessible web resource having the list of lncRNAs and circRNAs, which can be retrieved based on peptide length, sequence length and tissue-wise, and interaction between lncRNA-miRNA and miRNA-circRNA found in this study. This would be helpful to fellow researchers for augmenting related work in the crop.

## 2 Methods

### 2.1 Data collection

With the aim to perform a comprehensive study, a total of 53 raw RNA-Seq datasets (>1.2 billion reads) of black pepper were downloaded from the NCBI database. The dataset covered eight BioProjects, seven institutes across four countries, and six tissues (flower, fruit, leaf, panicle, root, and stem) from six black pepper cultivars *viz.* Reyin-1, Bragantina, Thottumuriyan, IPN No. LK-0-WU-0014181, panniyur-1, and Genotype 4226 (**Supplementary Table 1**).

### 2.2 Data pre-processing and transcriptome assembly

Raw reads obtained from NCBI were subjected to quality check using FastQC tool ver. 0.11.8 (Andrews, 2010), which helps us visualize various quality parameters like per base sequence quality, per base sequence content, presence of adaptor sequences, etc. The Trimmomatic ver. 0.39 (Bolger et al., 2014) software was then used to trim out the probable contaminants like adaptor sequences and low-quality reads with a Phred score of less than 30. The reference genome and annotation file of black pepper were downloaded from the GCGI (Group of Cotton Genetic Improvement, http://cotton.hszau.edu.cn/EN/index.php) website (Hu et al., 2019). The HISAT2-build command from HISAT2 ver. 2.2.0 (Kim et al., 2015; Pertea et al., 2015; Pertea et al., 2016) software was used to index the reference genome with splice sites and exons information retrieved from the annotation file. Index files were then used for aligning individual clean reads. Sam files obtained after the reads alignment were converted into binary bam files using Samtools software ver. 1.9 (Li et al., 2009), then the transcriptome assembly of the individual bam files was performed using StringTie ver 2.1.4 (Pertea et al., 2015) software to give out gtf files for each of the reads. Individual files corresponding to each tissue were then merged using StringTie–merge to get a single gtf (Gene Transfer Format) file for each tissue.

### 2.3 Genome-wide identification of lncRNAs in black pepper

**Figure 1:**
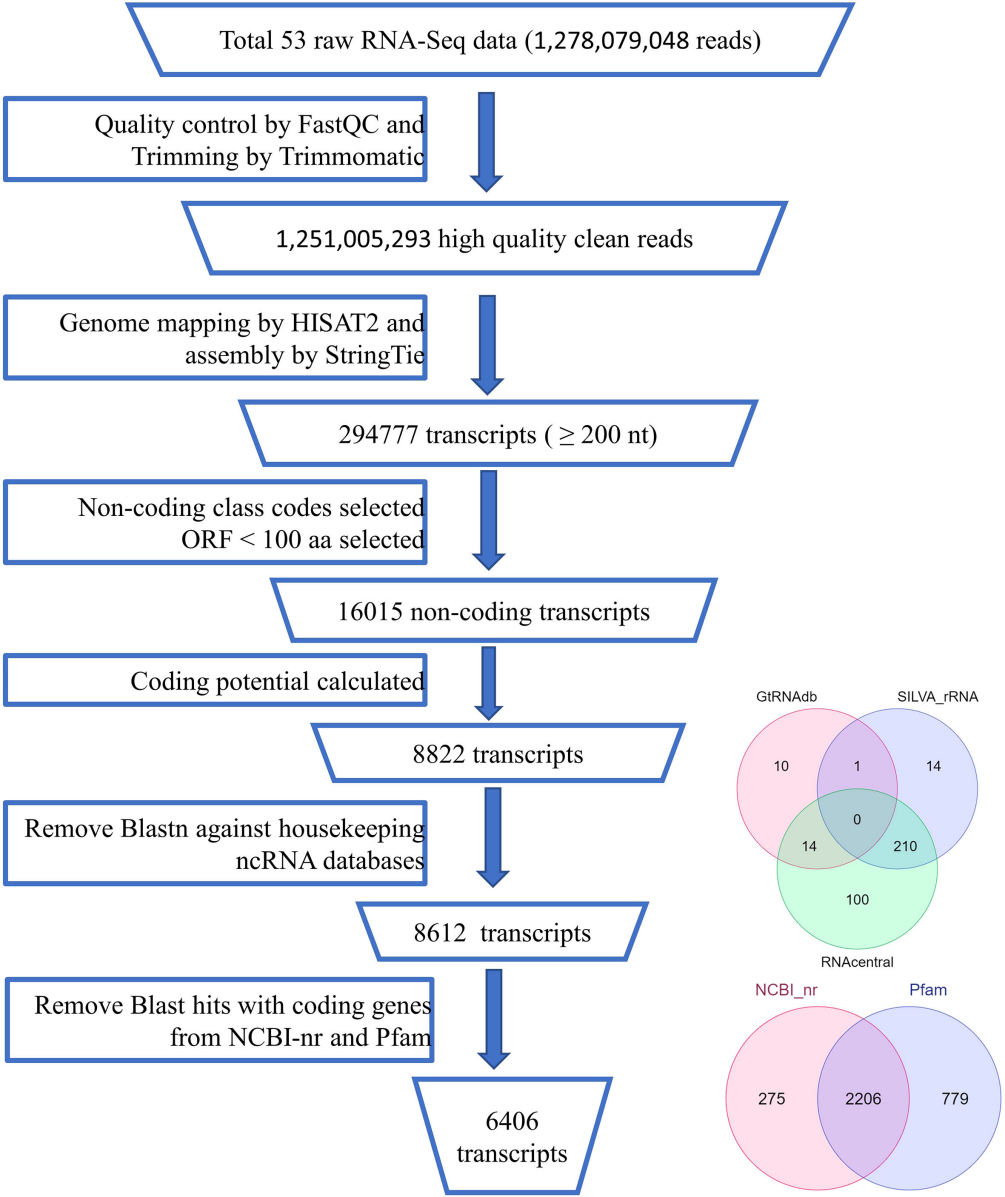
Pipeline of lncRNA identification.

Identification of lncRNA candidate transcripts from the assembled transcripts involved various steps as shown in **Figure 1**. First the fasta sequences corresponding to each transcript in the merged assembly file were extracted from their respective reference genome fasta files using the gffread program ver 0.12.3. As lncRNAs are RNA transcripts longer than 200 base pairs, using in-house Perl scripts, transcripts shorter than 200 bp were removed. Studies have shown that lncRNAs in general have a lower quality shorter ORFs than the protein-coding mRNAs. ORFPredictor (Min et al., 2005) was used to predict the ORFs in each transcript and those having ORFs longer than 300 nucleotides were removed. Binary classifiers like CPC2 ver 1.0.1 (Kang et al., 2017) and PLEK (Li et al., 2014) were used to classify the remaining sequences into coding or non-coding. Transcripts showing coding labels in all of them were discarded and kept as coding transcripts. The remaining transcripts may contain some housekeeping RNAs like rRNAs, tRNAs, snoRNAs, and other ncRNAs. So to get novel ncRNAs we did a BlastN search against the databases like SILVA database (https://www.arb-silva.de/download/arb-files/release138.1), GtRNAdb (http://gtrnadb.ucsc.edu/release18.1), and RNACentral (https://rnacentral.org/release16.0) and removed the transcripts showing >=95% identity (Sharma et al., 2017). Transcripts matching any reported proteins or protein families were identified and removed using Blast against NCBI-nr protein and Pfam (http://pfam.xfam.org/release33.1) databases (Altschul et al., 1990).

### 2.4 Conservation analysis by comparison with known plant lncRNAs in CANTATAdb

LncRNAs are poorly conserved compared to protein-coding mRNAs across species. Yet to check the conservativeness of black pepper’s lncRNAs, reported lncRNAs of 38 plant species available at the CANTATAdb ver. 2.0 (http://cantata.amu.edu.pl/) (Szcześniak et al., 2019) database were downloaded and a local BlastN search was performed against our identified black pepper lncRNAs as a query with 10^-20^ e-value as the cutoff.

### 2.5 LncRNA and circRNA characterization and functional annotation

#### 2.5.1 LncRNAs acting as a precursor of mature miRNAs

Plant cells contain small non-coding RNA transcripts miRNAs endogenously, which can bind to mRNAs and suppress their expression in the cell. These miRNAs are derived from longer primary miRNA transcripts which are converted to comparatively shorter pre-miRNAs (precursor) (Paraskevopoulou and Hatzigeorgiou, 2016). Finally, 18–24 nucleotide long mature miRNAs are produced by endonuclease action upon the pre-miRNAs. These lncRNAs can also act as a source for the biogenesis of mature miRNAs. Identified lncRNAs were matched against precursor miRNAs from the miRBase database using the BlastN software to find such black pepper lncRNAs which can act as a precursor miRNA.

#### 2.5.2 Identification of lncRNAs acting as target mimics of miRNAs

To find out the miRNAs, which may use the identified black pepper lncRNAs as their target mimics, the psRNATarget (Dai et al., 2018) (http://plantgrn.noble.org/psRNATarget/) server was used with black pepper miRNAs (Ding et al., 2021) and lncRNAs as inputs in the options of small RNA sequences and target sequences. Matches with stringent criteria of expectation ≤2 and allowed maximum energy to unpair the target site (UPE) = 25 were considered significant for our study.

#### 2.5.3 Identification of mRNA targets of identified miRNAs and their annotation

For creating the lncRNA-miRNA-mRNA network, mRNA targets of the identified miRNAs are to be found. PsRNATarget was run with identified miRNA sequences and black pepper cDNA sequences as input. Matches with expectation ≤2 and UPE ≥25 were considered significant. Identified mRNAs were annotated using OmicsBox (https://www.biobam.com/omicsbox/) software and GO terms obtained were used to functionally characterize the co-expressed lncRNAs. REVIGO (http://revigo.irb.hr/) server was used to further analyze the GO terms by summarizing the GO terms present and provides a graph based visualization of the GO terms.

#### 2.5.4 Identification of black pepper circRNAs

**Figure 2:**
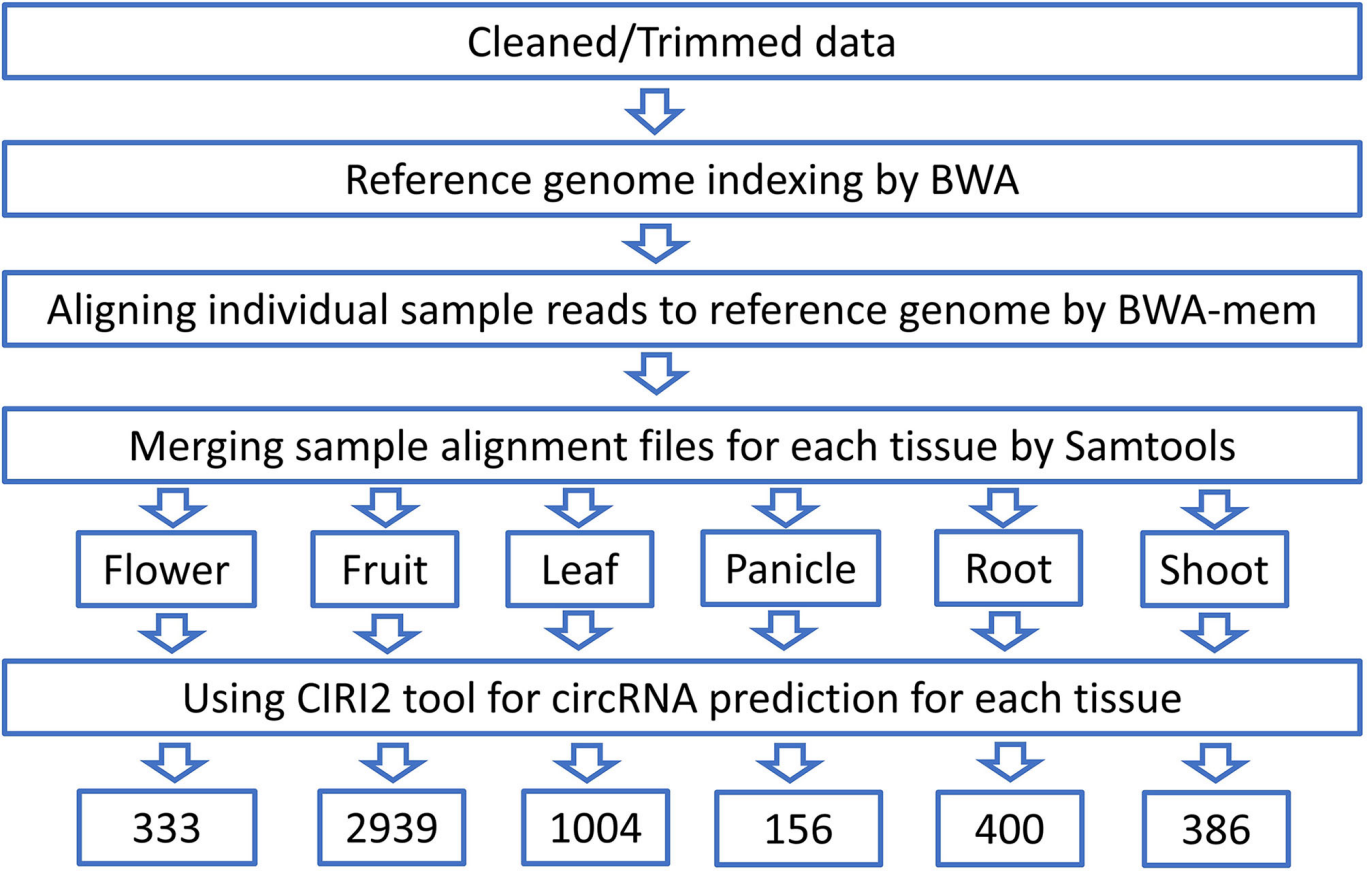
Schematic diagram of circRNA identification.

CircRNA identification pipeline starts from obtaining the clean reads after trimming out the low-quality bases and adaptor sequences. Reads retained from each sample were aligned to the reference genome of black pepper by BWA software ver. 0.7.17 (BWA mem–T 19) after creating an index using BWA–index module. Aligned sam files corresponding to each tissue were merged using Samtools ver. 1.14. The merged alignment files of each tissue were then provided to the CIRI2 ver.2.0 (Gao et al., 2018) tool as input for circRNA prediction. Novel circRNA were identified by comparing the identified circRNA with plant circRNA in PlantcircBase database (http://ibi.zju.edu.cn/plantcircbase/, release 7) (**Figure 2**).

#### 2.5.5 CircRNAs as miRNA sponges and miRNA-circRNA-mRNA relationship study

For understanding the relationship of the network between the miRNA, circRNA, and mRNA, previously identified and reported black pepper miRNAs by Ding et al. (2021) were collected and used. The circRNA targets of the miRNAs were found using TargetFinder software (Fahlgren and Carrington, 2010). The possible circRNA targets of the miRNAs were found by command line software TargetFinder. After this, the mRNA targets of the identified miRNAs were identified using the webserver psRNAtarget with miRNA sequences as small RNA sequences and CDS (coding sequence) of black pepper as the target sequences.

### 2.6 Black pepper lncRNA web-resource development

A web-resource, a Black pepper ncRNA database *BPncRDB* was created using the three-tier architecture, *viz*., client, middle, and database tiers that house all the results of this study related to the lncRNAs, circRNAs and their interactions with the miRNAs. The database was developed in MySQL database (https://www.mysql.com/) while the web-interface was prepared in PHP (https://www.php.net/) and HTML while designing was done using CSS and made dynamic using JavaScript. It was hosted on Apache server (https://httpd.apache.org/). XAMPP framework was used to design and deploy the webpage. The user can retrieve data as: (a) request from user to webserver, (b) query sent to MySQL database, (c) response generated by database and sent to web-interface, and (d) response of web-server to user. *BPncRDB* includes information of tissue-specific lncRNAs and circRNAs and their relationship with miRNAs, interaction between lncRNA-miRNA and miRNA-circRNA, etc.

## 3 Results

### 3.1 Data pre-processing and transcriptome assembly

The library was sequenced using the Illumina HiSeq X platform and 1,278,079,048 raw reads in 16 samples were collected. After discarding the Illumina platform’s adaptor sequences and other low-quality reads using the Trimmomatic software, we obtained 1,251,005,293 (97.88%) clean reads (**Supplementary Table 2**). The trimmed clean reads were aligned to black pepper’s reference genome using HISAT2 and approximately 70%–94% of reads were mapped across the 53 samples (**Supplementary Table 2**). StringTie software was then used to assemble the mapped reads of individual samples with respect to the reference annotation file of black pepper. Individual assembly files for each tissue were merged using the StringTie-merge module in order to get tissue-wise lncRNAs, and 294,777 transcripts were obtained.

### 3.2 Identification of long non-coding RNAs in black pepper

**Figure 3:**
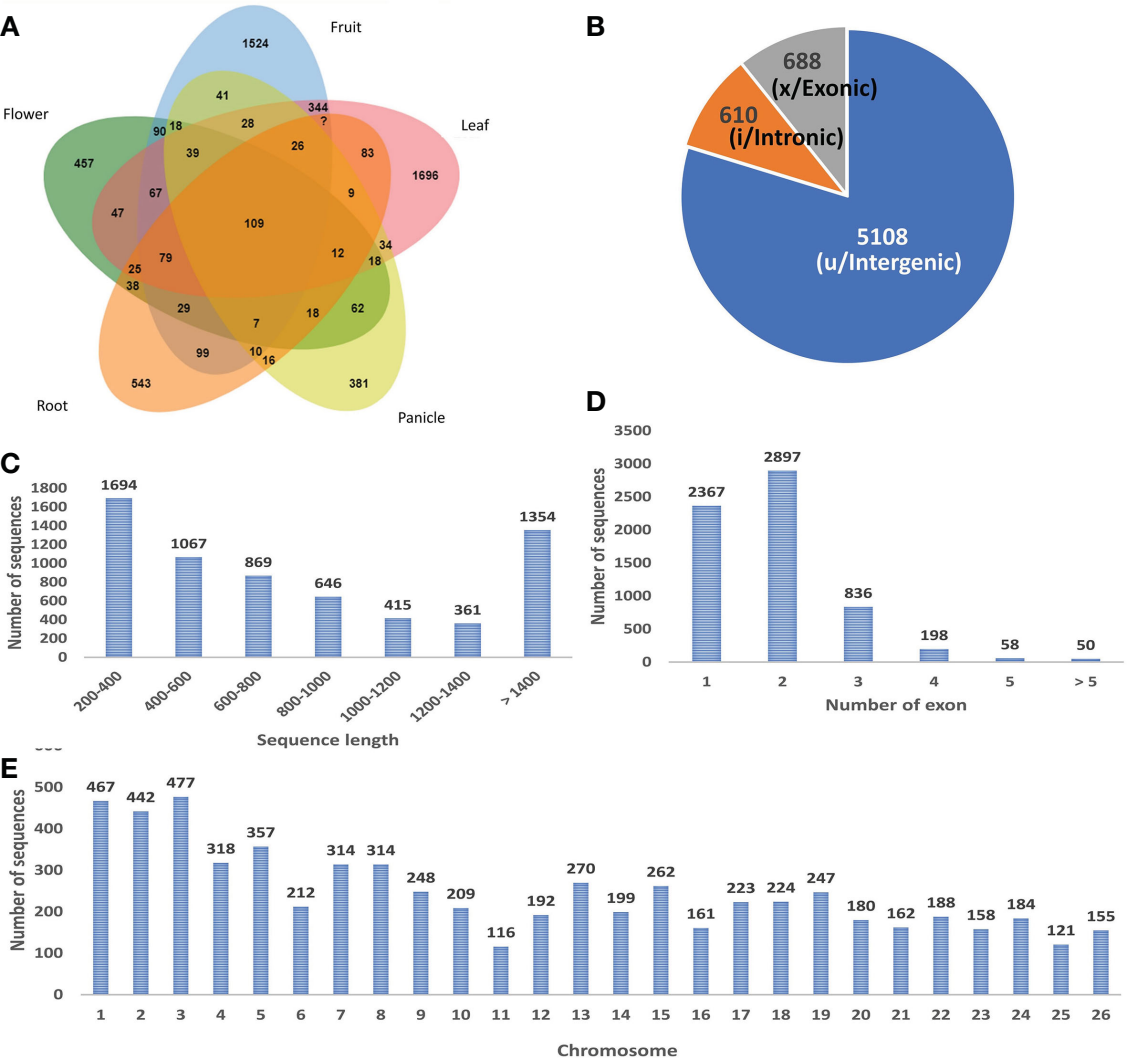
**(A)** Tissue-wise distribution of black pepper lncRNAs. **(B)** Classes of identified lncRNAs. **(C)** Length distribution of lncRNAs. **(D)** Exon distribution of lncRNAs. **(E)** Chromosome-wise distribution of identified lncRNAs.

A stringent filtering pipeline for the assembled transcripts was developed and used for the identification of those transcripts which fulfill the criteria of lncRNAs (**Figure 1**). GffCompare program was used to compare the four GTF files with an annotation file of black pepper and annotate the transcripts corresponding to their location on the genome with respect to the known genes (Pertea and Pertea, 2020). Out of the 15 class codes, i (intronic), u (intergenic), and x (natural antisense transcripts) represent the most probable non-coding transcripts and were selected for the downstream analysis, and the rest were discarded. Gffread was used to extract fasta sequences corresponding to the class codes and a total of 41090 sequences were found. In-house developed Perl scripts were used to remove sequences smaller than 200 nucleotides. To remove the potentially coding transcripts, ORFpredictor was used to find out the ORFs in each transcript and those having an ORF length >300 nucleotides were discarded. The coding probability of the remaining transcripts was calculated using CPC2 and PLEK software and taking the intersection of the results. Transcripts with CPC2 score >0.5 and predicted coding by PLEK were considered coding and discarded. Housekeeping RNAs were removed by BlastN against ncRNA databases, *viz*., Silvadb, gtRNAdb, and RNACentral and having percent identity >95%. Transcripts having similarity matching with any of the protein families in Pfam or genes in the NCBI-nr database were removed using BlastX (e-value 10^-3^). Finally, we identified 6406 novel black pepper long non-coding RNAs in black pepper, out of which 1115, 2621, 2727, 828, 1214, and 1003 were expressed in flower, fruit, leaf, panicle, root, and stem tissues, respectively (**Figure 3A**; **Supplementary Table 3**).

### 3.3 Characterization of identified black pepper lncRNAs

**Figure 4:**
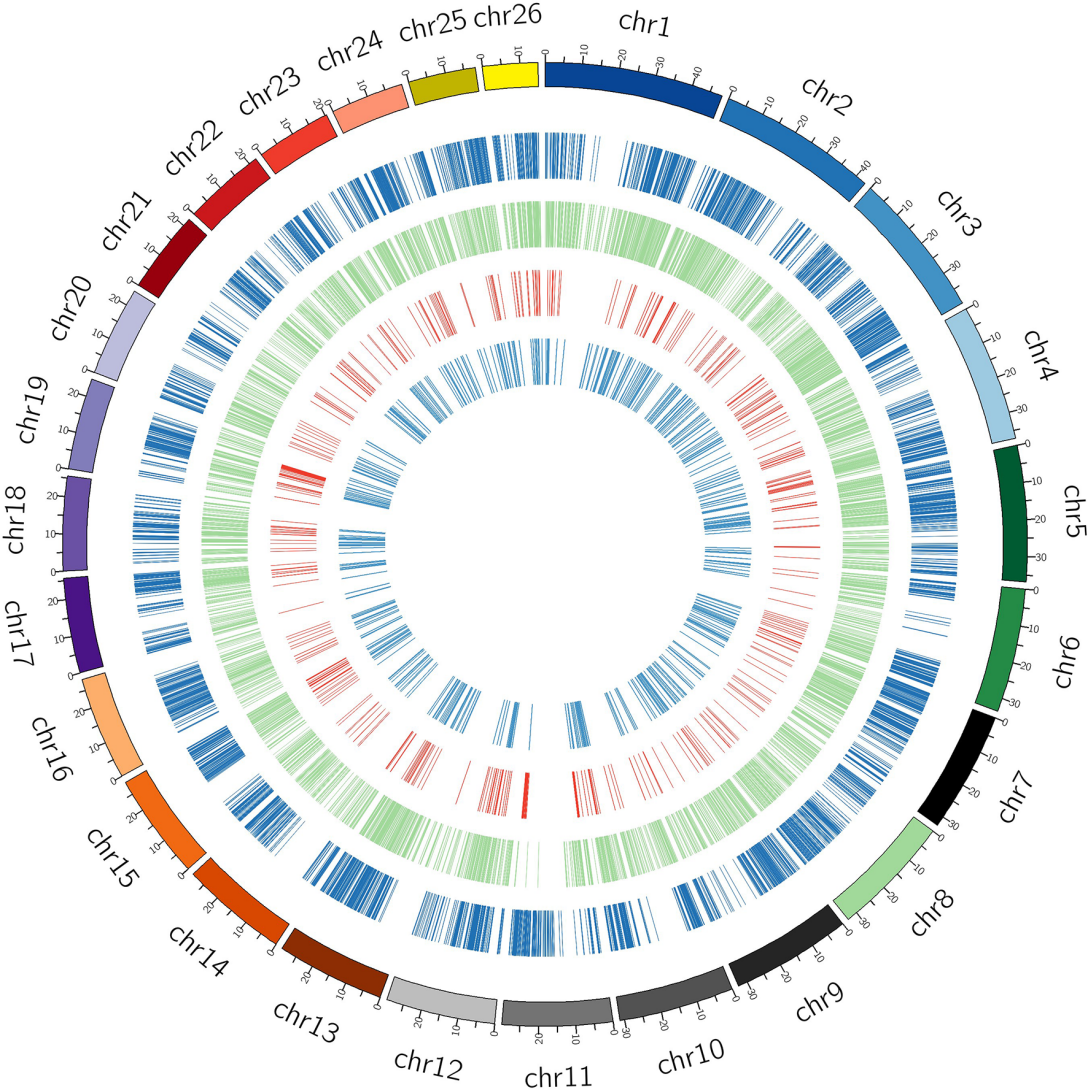
Black pepper circRNA/lncRNA shown chromosome wise. Outermost (dark blue) circle represents the black pepper circRNA, green circle represents intergenic lncRNAs, red circle represents intronic lncRNAs, and inner light blue circle represents antisense lncRNAs.

The study presents the first ever identification and characterization of black pepper lncRNAs to understand the functional importance of lncRNAs *via* various mechanisms affecting the crop’s yield and stress responses. In consistency with the previous lncRNA studies most (~80%) of the identified black pepper lncRNAs are intergenic in nature (**Figure 3B**). The length of the identified lncRNAs was distributed in the range of 200–10667 nucleotides which is much shorter than mRNAs which ranges up to 16.6 mega bases in length. More than 80% of the lncRNAs were shorter than 1500 nucleotides while only 10% were longer than 2000 nucleotides (**Figure 3C**). With an average of 1.88 exons per lncRNA, a major proportion (84.85%) of the lncRNAs in all six tissues were derived from 1 or 2 exons while only 1.68% were from ≥5 exons (**Figure 3D**). Chromosomal distribution of the identified lncRNA was depicted in (**Figure 3E**) and further visualized using the Circos software (**Figure 4**).

### 3.4 Conservation analysis of identified black pepper lncRNAs

To check on the conservation level of identified black pepper lncRNAs, BlastN against the previously known and reported lncRNAs of 38 plant species was performed with 10^-20^ as e-value cutoff. Only 45 high-confidence matches were found where 42 identified black pepper lncRNAs matched with 27 database lncRNAs from 13 plant species with a maximum of five matches to *Oryza barthii* and four matches to *Oryza rufipogon* and *Medicago truncatula* each (**Supplementary Table 4**). This result agrees with the literature suggesting the very poor conservation levels of lncRNAs compared to protein-coding mRNAs and are species- and tissue-specific.

### 3.5 Identified black pepper lncRNAs acting as miRNA precursors

**Figure 5:**
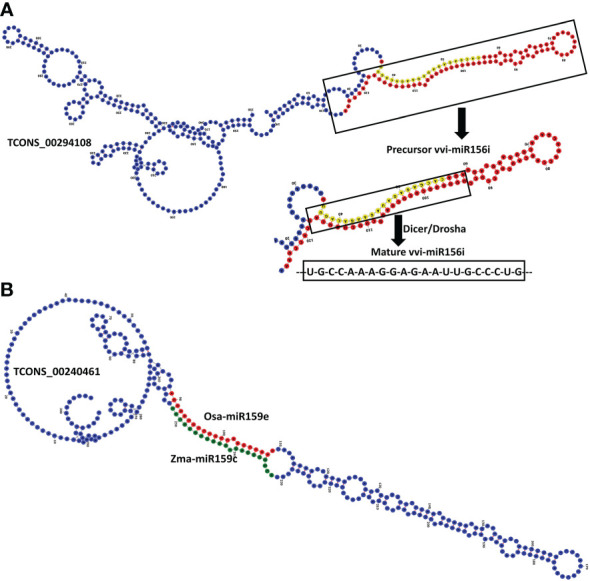
**(A)** RNAFold structure of lncRNA TCONS_00294108 showing the precursor (red) and mature (yellow) sequence of miRNA vvi-miR156i. **(B)** Structure of lncRNA TCONS_00240461 harboring the target sites of two miRNAs zma-miR159c and osa-miR159e.

lncRNAs are long RNAs present in the nucleus, nucleolus, and or cytoplasm and can function as the precursor for smaller ncRNAs such as snRNAs, snoRNAs, as well as the miRNAs (18–23 nts). To find out the black pepper lncRNAs which can possibly be precursors of known miRNAs, a BlastN search against the mirBase database was done (**Supplementary Table 5**). A total of 14 pre-miRNAs were matched >=90% with 36 unique lncRNAs, which suggests those lncRNAs can give rise to the mature miRNAs after being acted upon by nuclease enzymes like dicer and/or drosha. **Figure 5A** shows lncRNA TCONS_00294108 containing the precursor and mature sequences of miRNA vvi-miR156i.

### 3.6 Identification of lncRNAs and endogenous target mimics of miRNAs and analyzing lncRNA-miRNA-mRNA interaction network

**Figure 6:**
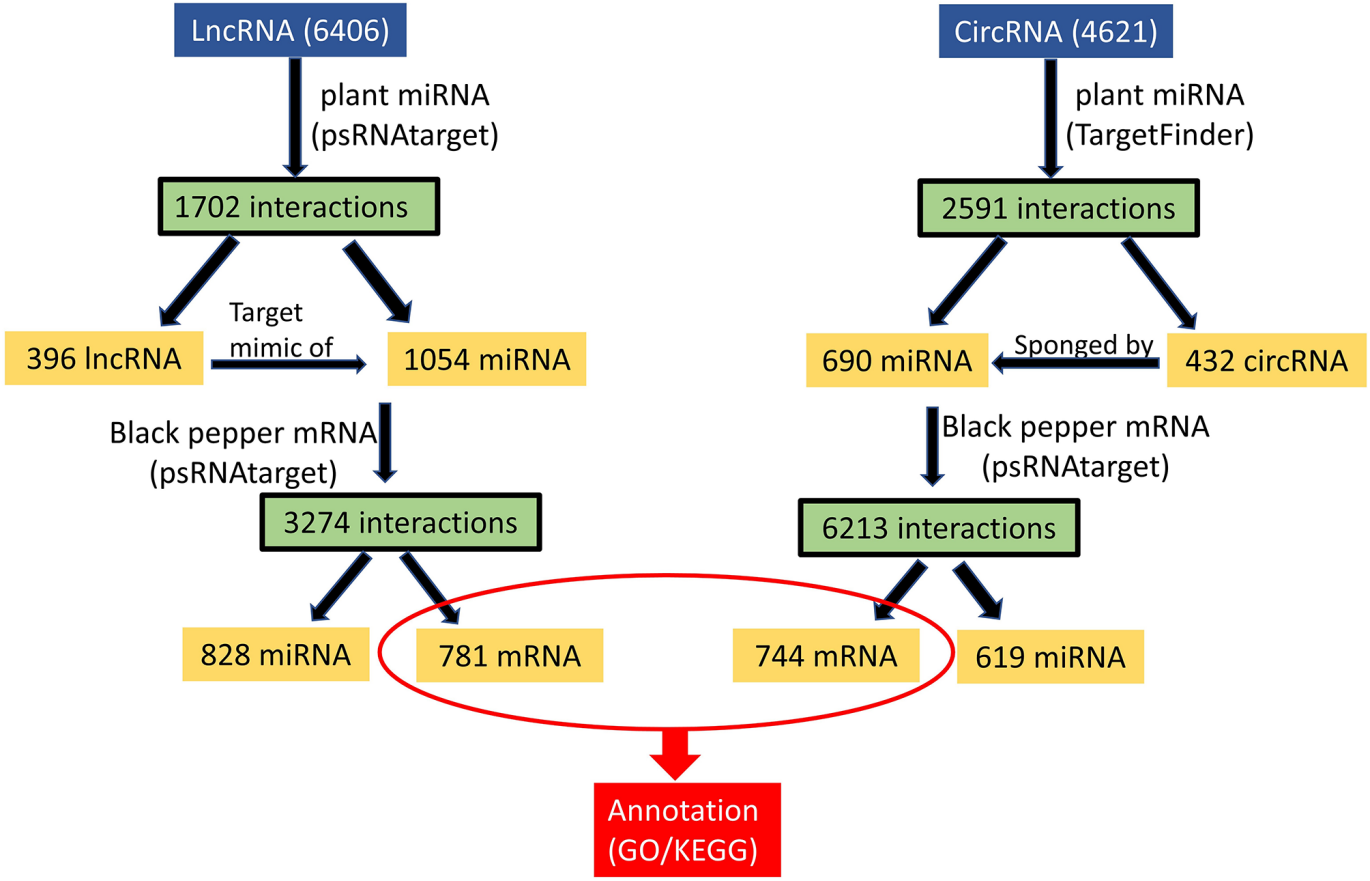
ceRNA analysis of lncRNA/circRNA.

**Figure 7:**
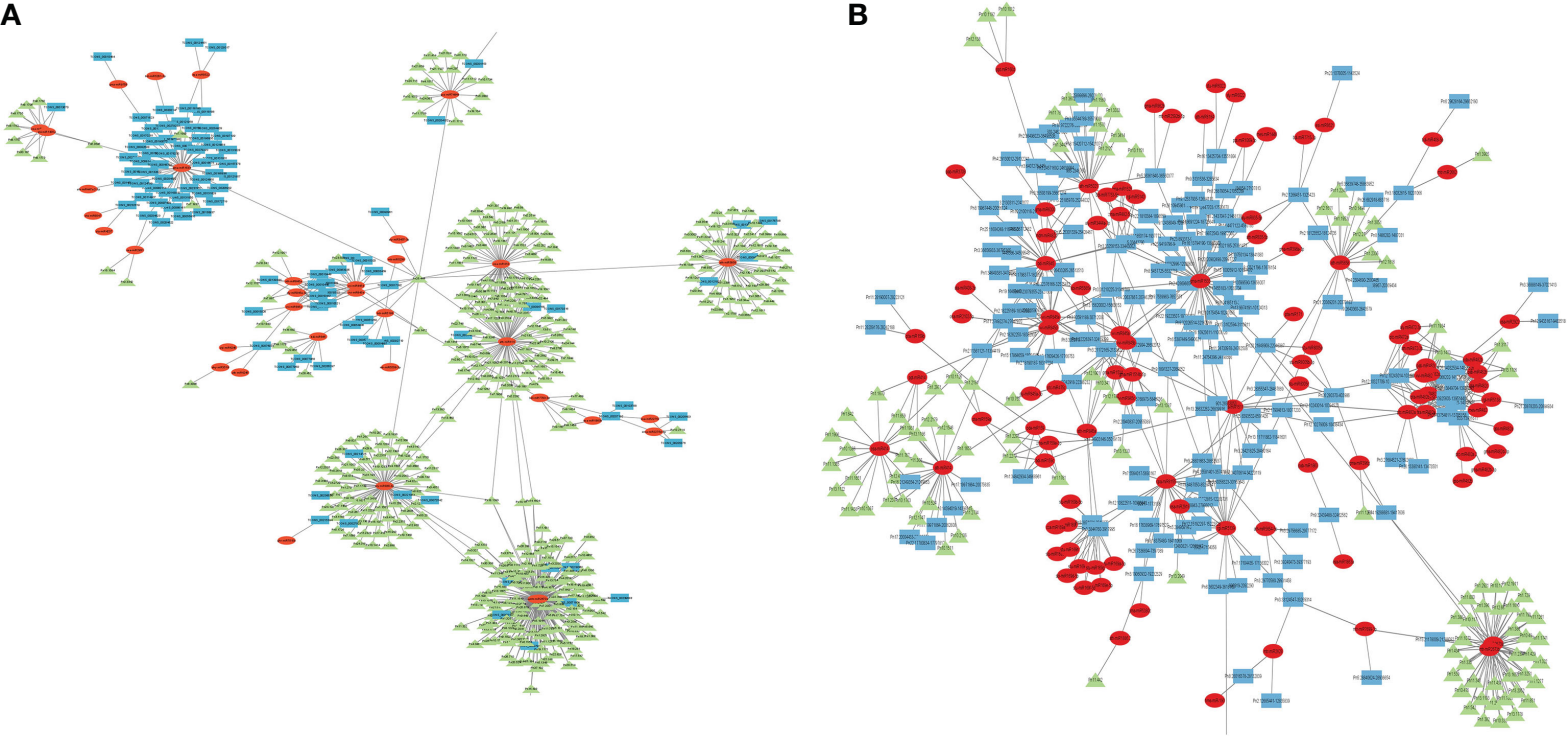
Cytoscape diagram showing the ceRNA network relationship between **(A)** lncRNA–miRNA–mRNA and **(B)** circRNA–miRNA–mRNA; green triangles representing mRNA, red ellipses representing miRNA, and blue rectangles representing lncRNA/circRNA.

**Figure 8:**
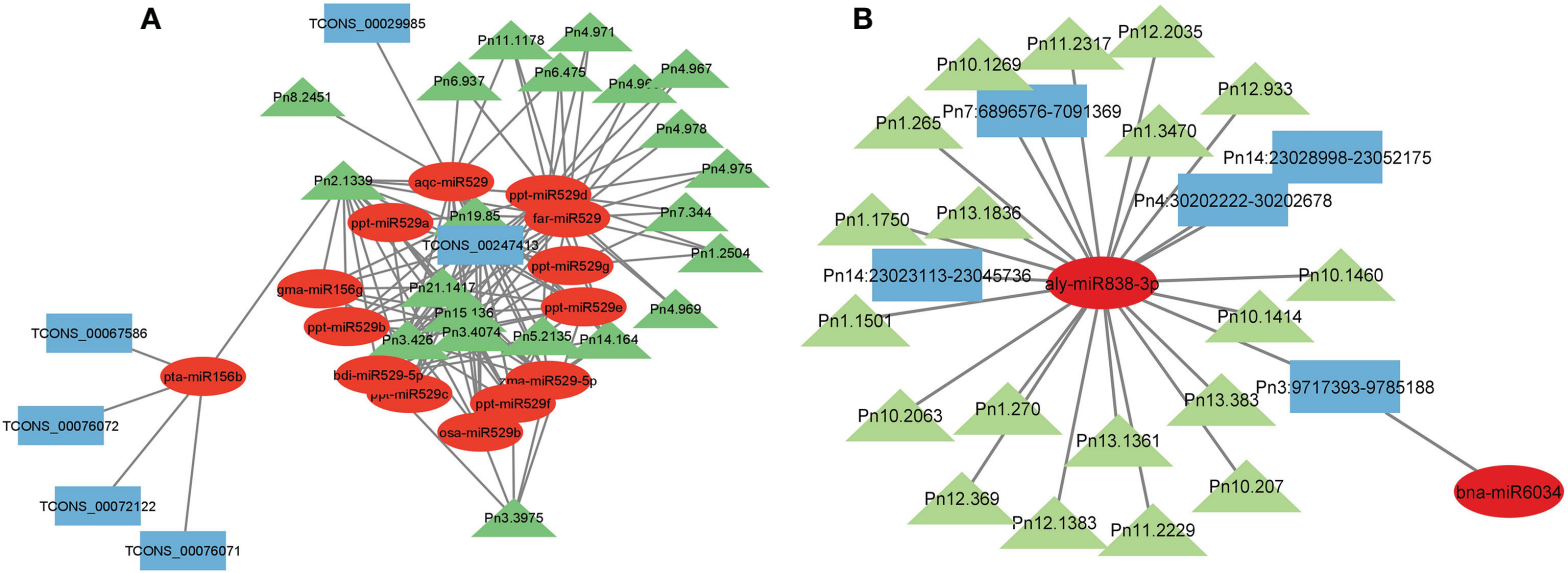
Representative figure showing the **(A)** lncRNA–miRNA–mRNA network **(B)** circRNA–miRNA–mRNA network.

Micro RNAs (miRNAs) are small ncRNAs (18–23 nts) which have the major function of mRNA expression regulation by binding the 3′ UTR of the protein coding mRNAs. The expression is suppressed or silenced depending upon the complementarity of the miRNA-mRNA binding. A full complementarity leads to mRNA degradation thus silencing while a partial binding decreases the mRNA expression level downregulating the genes. LncRNAs sometimes interfere in the process and act as a miRNA sponge and prohibit miRNA-mRNA binding. Identified lncRNAs and known plant miRNAs available at the psRNAtarget server were taken for this analysis. Identified black pepper lncRNAs were submitted to the psRNAtarget server as target sequences against the available plant miRNAs and run with default parameters of max UPE 25 and expectation ≤2 (**Supplementary Table 6**); 1702 interactions with 1054 unique miRNAs and 396 unique lncRNAs were found (**Figure 6**). **Figure 5B** shows the RNAFold structure of lncRNA TCONS00240461 (blue) with the binding sites of miRNAs Osa-miR159e (red) and Zma-miE159c (green). Target mRNAs of the identified miRNAs were also found using psRNAtarget by submitting black pepper CDS (coding sequence) as target and identified 1054 miRNAs as small RNAs (**Supplementary Table 7**); 3274 total interactions were found between the black pepper genes and miRNAs suggesting the possible gene regulations involved. Individual lncRNA-miRNA and miRNA-mRNA networks were merged and visualized using the Cytoscape software (**Figure 7A**), where we can see various networks involving miRNA and target mRNAs which can potentially be meddled by lncRNA thus regulating the normal gene regulation mechanism. For instance, miRNA pta-miR156b meant to be targeting mRNA Pn2.1339 is also capable of targeting four lncRNAs *viz*. TCONS_00067586, TCONS_00076072, TCONS_00072122, and TCONS_00076071. (**Figure 8A**).

### 3.7 Identification of black pepper circRNAs

**Figure 9:**
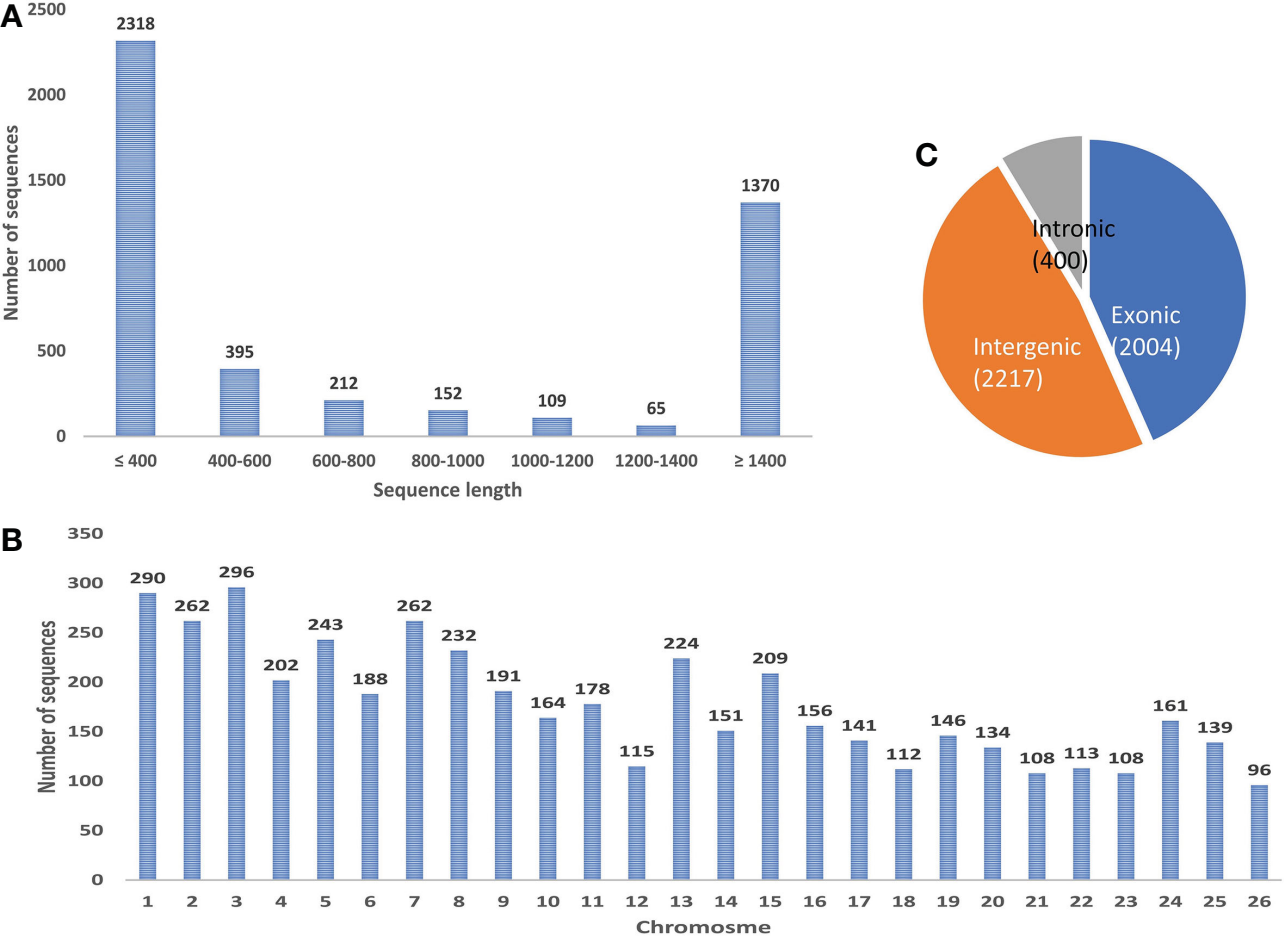
**(A)** Length-wise distribution of circRNA. **(B)** Chromosomal distribution of circRNA. **(C)** Class distribution of circRNA.

After removing the low-quality reads from the raw sequences, the clean reads (>1.2 billion reads) were aligned to the reference genome using BWA-mem generating 53 SAM files for each sample which were then merged corresponding to each of the six tissues and submitted to a reliable, sensitive, and widely used command line tool CIRI2. A total of 4621 distinct circRNAs with ≥2 backspliced reads were identified including 3871 novel circRNA. A total of 750 circRNAs were found to be overlapping with known plant circRNAs in PlantcircBase. Tissue-wise analysis revealed 333, 2939, 1004, 156, 400, and 386 circRNAs identified in the flower, fruit, leaf, panicle, root and stem tissues, respectively (**Supplementary Table 8**). Most the circRNAs were found to be ≤1000 nt (3077 circRNA smaller than 1000 nt) with a median length of approximately 400 nt, which is in consist with previous reports (**Figure 9A**) (Zheng et al., 2016). As described in previous studies, the abundance of circRNA was found to be lower than that of mRNA and lncRNA. Despite being widely distributed over all the chromosomes, genomic origin analysis showed most of the identified circRNA were coming from intergenic (47.98%) region followed by exonic (43.37%) regions and only few (8.66%) came from the intronic portion of the genome (**Figures 9B, C**).

### 3.8 Identification of circRNAs as sponges of miRNAs and analysis of the miRNA–circRNA–mRNA relationship

Various studies suggesting circRNAs can regulate gene expression by acting as ceRNAs for the miRNAs by competing and inhibiting the miRNA binding with the mRNA molecules. The result of TargetFinder revealed 2591 interactions where 690 unique miRNAs were found targeting 432 circRNAs in all tissues combined (**Supplementary Table 9**). CircRNA Pn4:30202222-30202678 was found to contain the putative miRNA binding site of aly-miR838-3p. mRNA targets of the identified miRNAs were identified using the psRNAtarget web server and a total of 6213 miRNA-mRNA interactions were found in which 619 unique miRNAs were targeting 744 unique black pepper mRNAs. Unique miRNA Pn3.1899 was found to be targeting 15 different mRNAs of black pepper (**Supplementary Table 10**). The miRNA–circRNA–mRNA network relationship was visualized using Cytoscape software (**Figures 7B**, **8B**).

### 3.9 Functional annotation of target genes of competitive endogenous lncRNAs and circRNAs

In this study, we have focused on the trans-regulating action of lncRNAs and circRNAs where they regulate the target gene function by sequestering the miRNAs thus acting as ceRNAs in the cell. A total of 781 and 744 mRNAs were found to have an interaction in 6406 lncRNAs and 4621 circRNAs. To know the functional significance of the identified target mRNAs, GO annotation was performed. Results of the GO analysis revealed important GO subcategories involved, for instance “protein modification process”, “transmembrane transport”, “anatomical structure development”, and “signaling” annotated in the biological process category of GO. Similarly, “transferase activity”, “catalytic activity”, and “oxidoreductase activity” were annotated in the molecular function category and “nucleus”, “membrane”, and “plastid” in the cellular component category of GO. KEGG pathway analysis further revealed gene functions. Highly enriched pathways included the ras singaling pathway, diterpenoid biosynthesis, fatty acid biosynthesis, and plant–pathogen interaction. Both the GO and KEGG analyses strongly suggest the potential role of lncRNAs and circRNAs in diverse biological processes in the black pepper plant (**Supplementary Tables 11–14**).

### 3.10 Development of web-genomic resource for the black pepper lncRNAs and circRNAs

**Figure 10:**
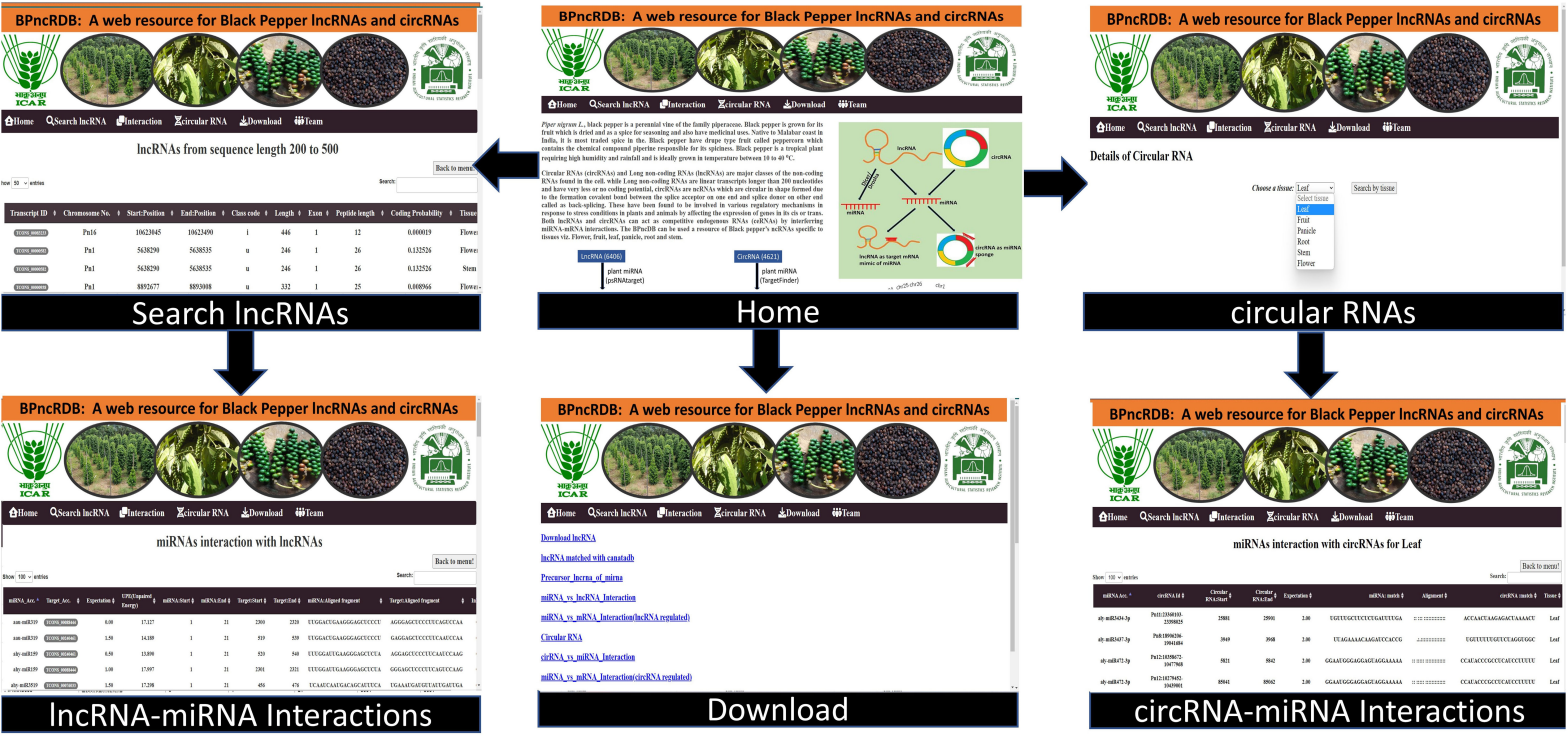
Layout of *BPncRDB* web-resource.

The black pepper non-coding RNA database, *BPncRDB*, is freely accessible at http://backlin.cabgrid.res.in/bpncrdb/index.php. The website features six tabs including Home, Search lncRNA, Interaction, circRNA, Download and Team (**Figure 10**). The database catalogues the black pepper’s 6406 lncRNAs and 4621 circRNAs along with the miRNAs’ interactions involved with them. The database also contains the information regarding the mRNA/genes involved in the ceRNA pathways with the lncRNA/circRNAs. All of the analyzed data that is available in the MySQL database can be downloaded using the links on the “Download” page.

## 4 Discussion

According to the ENCODE project, only 1%–2% of the human genome codes for proteins, and the vast majority of RNAs are non-coding RNAs such as tRNAs, rRNAs, microRNAs, lncRNAs, circRNAs, and others. LncRNAs and circRNAs are the most abundant non-coding RNAs present in the cell and have been recognized as the key regulators in genetic expression and are actively involved in the plant developmental stages and its response to biotic and abiotic responses. Understanding the functions of non-coding RNAs in biology has received more attention as a result of recent advancements in RNA sequencing technology, epigenomic methodologies, and computational prediction tools. LncRNAs/circRNAs are involved in a wide range of biological activities in all walks of life, which requires additional research (Quinn and Chang, 2016). Low levels of conservation of these ncRNAs between species make their characterization and functional annotation more challenging. Therefore, additional approach is required to create a distinct catalogue of lncRNAs/circRNAs based on numerous datasets for particular species such as black pepper for which there is no such study is available yet. In this study, a stringent methodology was used to identify 6406 lncRNAs and 4621 circRNAs from 53 RNAseq datasets, and they were then classified into three groups based on their position in respect to protein-coding genes. According to the earlier research, the lncRNAs found in this study differ from mRNAs in various unique ways, including having fewer exons, shorter transcript lengths, and lower conversation levels (Wang et al., 2019; Yan et al., 2020). It was found that most of the lncRNAs were between 200-800 base pairs in length and only a few over 2000 base pairs. A similar pattern was found in capsicum (Baruah et al., 2021). Although not evenly distributed, the identified black pepper lncRNAs were found to be distributed across all chromosomes. A similar trend has been reported in crops like rice, wheat and maize (Li et al., 2014; Wang et al., 2018; Lu et al., 2020). Identified lncRNAs belonged to the classes exonic, intergenic and intronic supporting the previous findings suggesting the genome-wide transcription of the lncRNAs (Sun et al., 2020; Baruah et al., 2021).

Studies reporting long non-coding RNAs that act as competitive endogenous RNAs (ceRNAs) have been published, for example, BLIL1 (blue light induced lncRNA), an *Arabidopsis thaliana* lncRNA, was found to be capable of reducing the hypocotyl elongation when subjected to blue light condition by having a competitive relationship with the miRNA miR167 and its mRNA target ARF6/8 (Sun et al., 2020). In rice, an intergenic lncRNA TCONS_00049880 regulates the expression of the SPL gene family by competitively binding to the miRNA osa-miR156. MiRNA-mediated gene regulation by the lncRNAs/circRNAs, also known as target mimicry, miRNA decoy, miRNA sponge, or competitive endogenous RNAs (ceRNAs) is one major interaction between ncRNAs and miRNAs. The first such case of lncRNA-miRNA binding was found in *Arabidopsis thaliana* where miRNA miR399 pairs with lncRNA IPS1 (Induced by Phosphate Starvation 1) leading to inability of miR399 to mediate PHO2 degradation. Similar cases of interactions were also found in our study, for instance, miRNA Osa-miR159e targets lncRNA TCONS_00240461 and sequesters the miRNA, hampering its probable functions in the cell (Meng et al., 2021).

CircRNA studies in plants have also revealed their role as miRNA sponges, where circRNAs with the miRNA binding sites attach and sequester the miRNAs thus regulating the expression of the miRNA targets (Fernandes et al., 2018). In Chinese cabbage circRNA A03:5084249|5089986 has the binding site of miRNA bra-miR5716 known for 15 target mRNAs possibly altering their functions including oxidation-reduction, ATPase activity, electron carrier activity, Myb-type HTH DNA-binding, and transmembrane transport (Wang et al., 2019). These interactions may alter the normal expression of mRNAs thus causing regulatory effect. Similar results were obtained in our study where identified black pepper circRNAs, for instance, miRNA aly-miR838-3p interacts with four circRNAs viz. Pn14:23023113-23045736, Pn7:6896576-7091369, Pn:30202222-30202678, and Pn:23028998-23052175, and is also targeting several mRNAs *viz*. Pn1.1750, Pn10.1460, Pn10.1414, etc., which are according KEGG analysis involved in processes like plant–pathogen interaction (ko04626), ribosome (ko03010), and carotenoid biosynthesis, respectively (ko00906). Plant cells endogenously produce small RNAs called miRNAs from larger precursor transcripts which can interact with the coding mRNAs causing partial transcriptional repression or complete silencing by cleavage, thus acting as a template (Fernandes et al., 2018). For example, lncRNA MSTRG.24217.2 of *Jatropha curcas* act as a precursor of miRNA miR396a which targets growth regulating factors (GRF) which controls the development of plant seeds (Yan et al., 2020). We found four such black pepper lncRNAs that can serve as the precursors for 20 miRNAs which could be involved in multiple cellular processes.

To date, there is no study on lncRNA/circRNAs and ceRNAs network analysis in black pepper. Here we identified genome-wide lncRNA and circRNA as well as explored the lncRNA-miRNA-mRNA and circRNA-miRNA-mRNA networks. BPncRDB, a comprehensive online database of black pepper lncRNAs and circRNAs accessible for academic and research around the world, would provide a platform to better understand the critical roles that these lncRNAs and circRNAs play in the growth and development as well as the responses towards biotic/abiotic stresses in black pepper.

## 5 Conclusion

With the goal of creating a comprehensive resource of black pepper lncRNA/circRNA (BPncRDB) and investigating their role as ceRNAs, 53 RNAseq datasets were downloaded by performing a comprehensive search of the publicly available repository NCBI and 6406 and 4621 lncRNAs and circRNAs were each identified using a stringent pipeline. Conservation analysis of the identified lncRNAs revealed 42 identified lncRNAs matching with the previously known plant lncRNAs in the database confirming the weakly conserved nature of lncRNAs. In addition to that 36 lncRNAs were also found to be acting as precursor of 14 miRNAs. A total of 744 and 781 genes were found to be in regulation network *via* lncRNA/circRNA-miRNA-mRNA pathways. Functional enrichment analysis of GO and KEGG revealed pathways such as diterpenoid biosynthesis, RAS signaling pathway, fatty acid biosynthesis, plant-pathogen interactions, etc., suggesting the potential role of lncRNA/circRNA in black pepper growth, development, and resistance against both biotic and abiotic stresses.

## Data availability statement

Data presented in the study are included in the article/**Supplementary Material**. Any further inquiries can be directed to the corresponding author/s.

## Author contributions

SJ and MI conceived and designed the study, BK and BS did the data curation and analysis. BK, BS, and UA were involved in database development. BK, BS, SJ and MI wrote the first draft of the manuscript. SJ, MI, UA and AR provided overall guidance and finalized the edited manuscript. All authors contributed to the article and approved the submitted version.
